# 

**Published:** 1866-06

**Authors:** 


### TO THE DENTISTS OF OHIO.

The entire Dental Profession of the State of Ohio are hereby cordially invited to meet in mass convention, in the city of Columbus, at Nautons Hall, on Tuesday and Wednesday, the 26th and 27th of June, to form a State Dental Society, and to devise and adopt such other measures as tend to elevate and advance the interests of the profession.

Come up, Brethren, and let us have such a meeting as our profession has not yet seen! The advantages to be derived from such an organization can not be overestimated. Other states around us are fully organized. Shall Ohio lag behind? *May* 21*st*, 1866.

C. N. Woodward, Ripley.
J. Taft, Cincinnati.
A. Berry, "
H. R. Smith, Cincinnati.
Wardel & James, "
Cameron & Wright "
H. A. Smith "
C. R. Taft "
S. W. Ludlow, Columbus.
John Fowler "
J. W. Wortman "
R. G. Warner, "
J. B. Beauman "
H. C. Fowler "
W. C. Lilley, Circleville.
M. DeCamp, Mansfield.
N. W. Williams, Xenia.
Geo. L. Paine, "
Geo. Watt, "
C. C. Carroll, Ravenna.
B. F. & D. Gibbons, Warren.
C. Palmer "
B. T. Spelman "
M. M. Oldham, Springfield.
D. Philips, "

A. E. Lyman, Garrettsville.
S. R. Bequith, Kindsman.
F. S. Whitslar, Youngstown.
J. R. Finney "
J. C. Whinery, Salem.
D. Herrick, Trumbell, Co.
A. A. Blount, Springfield.
J. Armstrong, Bucyrus.
A. W. Maxwell, Gallion.
Rhoads & Myers, Woster.
C. R. Butler, Cleveland.
B. F. Robinson "
B. Strickland "
F. S. Slosson "
L. Buffett "
Gibbs & Stout, Portsmouth.
H. H. Harrison, Cadiz.
J. W. Scott, "
H. Morrison, Steubenville.
O. Kells "
W. C. Anderson "
J. Chesebrough, Toledo.
C. H. Harroun, "
B. F. Rosson, Troy.
C. Bradley, Dayton.

Thos. K. Brewster, Dayton.

### MEASURING CUP.

This is a little appliance arranged by Dr. Shadoan, for determining the amount of rubber for any given case. By it the amount can be precisely determined, which in many cases is an important item. It consists of a small cup, with a glass tube and scale attached. The method of using it will be suggestive at first sight. We presume it can be obtained at any of the Dental Depots.

### AMERICAN DENTAL ASSOCIATION.

The annual meeting of this body will be held in Boston Mass., beginning on Tuesday the 31st of July. This will probably be the largest meeting of this Association ever held. Boston is certainly a very desirable place, on many accounts for holding the meeting. There are there many things of general interest, that always attract the attention of the visitor, and we doubt not, all members and delegates will be delighted to go. And in addition to this the number of local societies, entitled to send delegates, has considerably increased, and all the pre-existing societies have been rapidly growing, so that they are all entitled to a larger number of delegates than heretofore ; and every society so far as practicable have elected delegates who are not members of the National Association, and all the old members, will be induced by former experiences to be present. Now all this we regard as very good data upon which to anticipate a large meeting.

All the committees so far as we are advised are industriously engaged in the accomplishment of the work, assigned them at the last meeting, so that without doubt there will be much presented for the consideration of the Association. We would suggest that the association take into consideration the propriety of adopting a code of Ethics. Something of the kind, it seems to us, would be advantageous,—securing in the profession more uniformity, where there has hertofore been much diversity. We would not by this, be understood, as advocating the idea that the association should assume judicial power or authority; but that such a code might issue in an advisory aspect. This subject was touched upon in an article read before the last meeting, but the subject was not acted upon or discussed. We expect to enjoy the blessed privilege of a large and enthusiastic meeting.

### THE AMERICAN DENTAL CONVENTION.

### CENTRAL STATES DENTAL ASSOCIATION.

The Third Annual Meeting of the Central States Dental Association, will be held in the Lecture room of the Kentucky School of Medicine, in the City of Louisville, commencing on Tuesday, July the 17th, 1866, at 10 o'clock, A. M.

Your attendance is earnestly requested.

W. H. Shadoan, Sec'y.

SUBJECTS FOR DISCUSSION.—First. Temporary Teeth their importance and uses. Second. Indications for the Extraction of Teeth. Third. Material for Filling teeth. 1st. Gold:—Soft foil. 2d. Gold:—Adhesive foil. 3d. Gold:—Sponge. 4th. Other materials than Gold. Fourth. Alveolar Abscess. Treatment and Cure. Fifth. Mechanical Appliances—Materials. 1st. Gold, Rubber, Silver, &c. 2d. Utility of Temporary teeth. 3d. Permanent teeth. Sixth. Miscellaneous.

W. G. Redman, }

W. D. Stone, } Ex. Com.

AMERICAN DENTAL ASSOCIATION—Meets annually (this year at Boston,) *Pres.* C. W. SPALDING, of St. Louis, *Rec. Sec.* J. TAFT, of Cincinnati, *Cor. Sec.* L. D. SHEPARD, of Salem, Mass.

*List of Societies represented in the American Dental Association.*

ALBANY DENTAL ASSOCIATION—Meets monthly at Albany. *Pres.* ROBERT NELSON *Rec Sec.* W. F. WINNE, *Cor. Sec.* B. WOOD, of Albany.

BUFFALO DENTAL SOCIETY—Meets monthly at Buffalo. *Pres.* R. G. SNOW, *Rec Sec.* GEO. B. SNOW, of Buffalo.

BROOKLYN DENTAL ASSOCIATION—Meets semi-monthly *Pres.* W. C. PARKS, *Rec. Sec.* W. B. HURD, *Cor. Sec.* W. H. ATKINSON, of New York.

CINCINNATI DENTAL SOCIETY—Meets semi-monthly at Cincinnati. *Pres.* C. H. JAMES, *Rec. Sec.* WM. TAFT.

CENTRAL OHIO DENTAL ASSOCIATION—Meets Semi-annually* on the second Tuesday of May and November. *Pres.* Dr. M. DECAMP, Mansfield, O., *Sec.* A. W. MAXWELL Gallion, O.

CENTRAL STATES DENTAL ASSOCIATION —Holds two meetings. The Annual meeting at Louisville, Ky., during the Hollidays. The Semi-annual on the second Tuesday o April. *Pres.* Dr. W H. MORGAN, *Sec.* W. H. SHADOAN, Louisville, Ky.

CENTRAL NEW YORK DENTAL ASSOCIATION Meets semi-annually. *Pres.* S. B. PALMER, *Rec. Sec.* S. G. MARTIN, *Cor. Sec.* P. HARRIS of Skaneatles

CHICAGO DENTAL SOCIETY—Meets monthly at Chicago. *Pres.* J. W. ELLIS, *Rec. and* [illegible]*or. Sec.* E. W. SAWYER of Chicago.

CONNECTICUT VALLEY DENTAL ASSOCIATION—Meets semi-annually, second Tuesday of May and October, *Pres* J. BEALS. *Rec. Sec.* L. D. SHEPHERD, of Salem, Mass.

HUDSON VALLEY DENTAL ASSOCIATION—meets monthly at Troy, N. Y. *Pres.* H. H. YOUNG, *Rec. Sec.* S. J. ANDRES, *Cor. Sec.* S. P. WELCH, of Troy.

INDIANA STATE DENTAL SOCIETY—Meets annually at Indianapolis on the last Tuesday of June. *Pres.* A M. MOORE, *Rec. and Cor. Sec* JOS. RICHARDSON, of Terre Haute, Ind

IOWA STATE DENTAL SOCIETY—Meets semi-annually Jan. and July. *Pres.* L. C. INGERSOLL *Rec. Sec.* H. S. CHASE.

LEBANON VALLEY DENTAL ASSOCIATION—*Prest.* W. K. BRENIZER.

LOUISVILLE DENTAL SOCIETY—Meets on the first Tuesday of each month. *Pres.* Dr. N. CLUTE, *Cor. Sec.* W. H. SHADOAN.

MAD RIVER VALLEY DENTAL SOCIETY—Meets quarterly. Next meeting in Dayton, on the first Tuesday in June next. *Prest.*, M. M. OLDHAM, *Sect.*, J. BLAKE.

MICHIGAN DENTAL SOCIETY—Meets annually fourth Tuesday of Jan. at Detroit- *Pres.* J. W. FIELD, *Rec. and Cor. Sec.* G. W. STONE, Albion.

MISSISSIPPI VALLEY DENTAL ASSOCIATION—Meets annually in February at Cincinnati, *Pres.* G W KEELY, *Rec. Sec.* WM. TAFT, *Cor Sec.* H. A. SMITH, of Cin.

MISSOURI DENTAL ASSOCIATION—Meets on the first Tuesday of June, 1866, in St Louis *Pres.* Dr. H. J. MCKELLOPS. St. Louis, *Sec* H. JUDD, St. Louis.

MERRIMAC VALLEY DENTAL SOCIETY—Meets semi annually* first Tuesday of Oct. and May. *Pres.* A. LAWRENCE, *Rec. Sec.* G. A. GERRY, of Lowell.

NASHVILLE DENTAL SOCIETY—Meets on the second Tuesday of each month. *Pres.* W. H. MORGAN, *Cor. Sce.* J. C. RUSSELL.

NEW YORK SOCIETY OF DENTAL SURGEONS—Meets semi-monthly in New York, *Pres.* W. H. ALLEN, *Rec. Sec.* C. D. ALLEN, *Cor. Sec.* C. P. FITCH, of New York.

NEW HAVEN DENTAL SOCIETY—Meets on the first Wednesday of each month at New Haven. *Pres.* J. T. METCALF, *Rec and Cor. Sec.* C. L. SMITH, of New Haven.

NEW LONDON COUNTY DENTAL SOCIETY—Meets monthly.* *Pres.* R. W. BROWNE *Rec. Sec.* WM. ALLENDER.

NORTHERN OHIO DENTAL ASSOCIATION—Meets Semi-annually, (first Tuesday of May and October.) in Cleveland. *Pres.* B. STRICKLAND, *Rec. Sec.* L. BUFFETT, *Cor. Sec* B. F. ROBINSON, Cleveland.

OHIO DENTAL COLLEGE ASSOCIATION—Meets annually in February at Cincinnati. *Pres.* J. H. MCKELLOPS, *Rec. Sec.* J. TAFT, of Cincinnati.

ODONTOGRAPHIC SOCIETY OF PENNSYLVANIA—Meets monthly in Philadelphia *Pres.* JAS. N. HARRIS, *Rec. Sec.* R. J. HORNER, *Cor. Sec.* J. H. MCQUILLEN, of Phila

**PENNSYLVANIA ASSOCIATION OF DENTAL SURGEONS**—Meets monthly in Phila *Pres.* W. W. FOUCHE, *Rec. Sec* JAMES TRUMAN *Cor. Sec.* G. W. ELLIS, of Philadelphia.

**PITTSBURG DENTAL ASSOCIATION**—Meets monthly, (second Tuesday, in Pittsburg *Pres.* M. E. GILLESPIE, *Rec. Sec.* J. D WHITE.

**PENINSULAR DENTAL SOCIETY**—Meets quarterly.* *Pres.* SAMUEL MARSHALL, *Rec. Sec.* W. G. A. BONWILL, *Cor. Sec.* S. S. NONES.

**WESTERN DENTAL SOCIETY**—Meets annually.* *Pres.* T. F. ABELL, *Rec. Sec.* C. W. SPALDING, *Cor. Sec.* E. A BOGUE.

**ST. LOUIS DENTAL SOCIETY**—Meets monthly at St. Louis.

**WESTERN NEW YORK DENTAL ASSOCIATION**—Meets semi-annually, *(next meeting at Lockport,) *Pres.* GEO. E. HAYES, *Sec* GEO. B. SNOW.

**ST. LOUIS ODONTOLOGICAL SOCIETY**—*Pres.* E. HALE, *Rec. Sec.* G. H. SILVERS, *Cor. Sec.* G. G. SAMUEL.

**AMERICAN DENTAL CONVENTION**—Meets annually first Tuesday of August, (nex meeting at New York,) *Pres.* H. E. PEEBLES, *Rec. Sec.* H. A. SMITH. *Cor. Sec.* W. H. ALLEN, of New York.

**Place of meeting fixed by appointment.*

### CONTRIBUTORS.

W. H. ATKINSON, M. D., D. D. S.

B. WOOD, M. D.

W. H. SHADOAN.

WM. A. PEASE, D. D. S.

LEON F. HARVEY, M. D.

H. A. SMITH. D. D. S.

GEO. B. SNOW, D. D. S.

WM. O. KULP.

A. S. TALBERT, D. D. S.

JOS. RICHARDSON, D. D. S.

A. LAWRENCE.

H. S. CHASE, D. D. S.

R. J. POORE.

GEO. L. PAINE, D. D. S.

A A. BLOUNT, M. D.

G. A. MILLS.

C. W SPALDING, D.D.S.

J. DOUGLASS.

J. S. LATIMER, D. D. S.

J. D. ANDERSON, D. D. S.

H. BENEDICT, D. D. S.

S. D. STEWART.

W. H GATES.

A. M. LESLIE, D. D. S.

GEO. WATT, D. D. S.

J. TAFT, D. D. S.

S. B. PALMER.

F. ABBOTT.

A. BERRY, D. D S.

A. W. MAXWELL.

H. F BISHOP.

JOSEPH S. HILDRETH, M. D

GAM'L JACKSON.

H. T. CRESSINGER.

J. CHESEBROUGH, D. D.

G. W. REDMAN.

### EXCHANGES,

ST. LOUIS MEDICAL AND SURGICAL JOURNAL,
BOSTON MEDICAL AND SURGICAL JOURNAL,
THE PACIFIC MEDICAL AND SURGICAL JOURNAL,
BUFFALO MEDICAL AND SURGICAL JOURNAL,
OHIO MEDICAL AND SURGICAL JOURNAL,
PHILADELPHIA MEDICAL AND SURGICAL JOURNAL,
WESTERN LANCET AND OBSERVER,
CHICAGO MEDICAL JOURNAL,
DENTAL COSMOS,
L' ART DENTAIRE,
THE LONDON DENTAL REVIEW,
BRAITHWAITE'S RETROSPECT,
SAN FRANCISCO MEDICAL PRESS,
THE DENTAL TIMES,
LADIES' REPOSITORY,
HALL'S JOURNAL OF HEALTH,
DEUTSCHE VIERTELJAHRSSCHRIFT,
DENTAL CIRCULAR AND EXAMINER,
CHICAGO MEDICAL EXAMINER.
THE CINCINNATI JOURNAL OF MEDICINE.
MEDICAL RECORDER.
XENIA TORCHLIGHT.
THE U. S. MEDICAL AND SURGICAL JOURNAL.
THE DENTAL QUARTERLY.

### CONTENTS.

American Dental Association,........ 282

The American Dental Convention........ 282

Central States Dental Association,........ 283

Automatic Plugger,........ 283

Bradley's Lip and Cheek Holder,........ 284

A File Carrier,........ 284

### The Dental Register

### OF THE WEST.

*Issued Monthly, at $3 a Year in Advance.*

DEVOTED TO THE INTERESTS OF THE DENTAL PROFESSION.

**EDITED BY J. TAFT**

The volume commences in January and closes in December.

The Register being issued monthly is always fresh, therefore indispensable to the practitioner who desires to keep posted up with the new inventions and improvements which are daily developed. Neither expense nor effort will be spared to give value and variety to its contents. Articles will be illustrated with well executed engravings whenever they will add to the interest or assist in explanation.

Its extensive circulation and frequency of issue makes it one of the BEST DENTAL ADVERTISING MEDIUMS in the United States.

RATES OF ADVERTISING.

One page, 1 year, $50. Half page, 1 year, $30. Quarter page, or card, 1 year $20.

All advertising bills payable in advance, or at furthest after the issue of the first number containing the advertisement. All transient advertisements to be paid for in advance.

☞ CONTRIBUTIONS SOLICITED.

Address, J. TAFT, or "DENTAL REGISTER," Cincinnati, Ohio.

Business Communications to

**J. TAFT, Publisher,**
No. 172 Race Street.

